# A COVID moonshot: assessment of ligand binding to the SARS-CoV-2 main protease by saturation transfer difference NMR spectroscopy

**DOI:** 10.1007/s10858-021-00365-x

**Published:** 2021-04-15

**Authors:** Anastassia L. Kantsadi, Emma Cattermole, Minos-Timotheos Matsoukas, Georgios A. Spyroulias, Ioannis Vakonakis

**Affiliations:** 1 Department of Biochemistry, University of Oxford, South Parks Road, Oxford, OX1 3QU UK; 2 Department of Pharmacy, University of Patras, Panepistimioupoli Campus, 26504 Patras, Greece

**Keywords:** SARS-CoV-2, COVID-19, M^pro^, NMR, STD, Molecular dynamics

## Abstract

Severe acute respiratory syndrome coronavirus 2 (SARS-CoV-2) is the etiological cause of the coronavirus disease 2019, for which no effective antiviral therapeutics are available. The SARS-CoV-2 main protease (M^pro^) is essential for viral replication and constitutes a promising therapeutic target. Many efforts aimed at deriving effective M^pro^ inhibitors are currently underway, including an international open-science discovery project, codenamed COVID Moonshot. As part of COVID Moonshot, we used saturation transfer difference nuclear magnetic resonance (STD-NMR) spectroscopy to assess the binding of putative M^pro^ ligands to the viral protease, including molecules identified by crystallographic fragment screening and novel compounds designed as M^pro^ inhibitors. In this manner, we aimed to complement enzymatic activity assays of M^pro^ performed by other groups with information on ligand affinity. We have made the M^pro^ STD-NMR data publicly available. Here, we provide detailed information on the NMR protocols used and challenges faced, thereby placing these data into context. Our goal is to assist the interpretation of M^pro^ STD-NMR data, thereby accelerating ongoing drug design efforts.

**Supplementary Information:**

The online version contains supplementary material available at 10.1007/s10858-021-00365-x.

## Introduction

Infections by the severe acute respiratory syndrome coronavirus 2 (SARS-CoV-2) resulted in approximately 1.8 million deaths in 2020 (WHO 2021) and led to the coronavirus 2019 (COVID-19) pandemic (Kucharski et al. 2020; Wu et al. 2020; Zhu et al. 2019). SARS-CoV-2 is a zoonotic betacoronavirus highly similar to SARS-CoV and MERS-CoV, which caused outbreaks in 2002 and 2012, respectively (Bermingham et al. 2012; Kuiken et al. 2003; Zaki et al. 2012). SARS-CoV-2 encodes its proteome in a single, positive-sense, linear RNA molecule of ~ 30 kb length, the majority of which (~ 21.5 kb) is translated into two polypeptides, pp1a and pp1ab, via ribosomal frame-shifting (Thiel et al. 2003; Bredenbeek et al. 1990). Key viral enzymes and factors, including the reverse-transcriptase machinery, inhibitors of host translation and molecules signalling for host cell survival, are released from pp1a and pp1ab via post-translational cleavage by two viral cysteine proteases (Hilgenfeld 2014). These proteases, a papain-like enzyme cleaving pp1ab at three sites, and a 3C-like protease cleaving the polypeptide at 11 sites, are primary targets for the development of antiviral drugs.

The 3C-like protease of SARS-CoV-2, also known as the viral main protease (M^pro^), has been the target of intense study owing to its centrality in viral replication. M^pro^ studies have benefited from previous structural analyses of the SARC-CoV 3C-like protease and the earlier development of putative inhibitors (Ghosh et al. 2007; Verschueren et al. 2008; Yang et al. 2003,2005). The active sites of these proteases are highly conserved, and peptidomimetic inhibitors active against M^pro^ are also potent against the SARS-CoV 3C-like protease (Zhang et al. 2020; Rut et al. 2020). However, to date no M^pro^-targeting inhibitors have been validated in clinical trials. In order to accelerate M^pro^ inhibitor development, an international, crowd-funded, open-science project was formed under the banner of COVID Moonshot (Achdout et al. 2020), combining high-throughput crystallographic screening (Douangamath et al. 2020), computational chemistry, enzymatic activity assays and mass spectrometry (El-Baba et al. 2020) among the many methodologies contributed by collaborating groups.

As part of COVID Moonshot, we utilised saturation transfer difference nuclear magnetic resonance (STD-NMR) spectroscopy (Mayer and Meyer 1999; Becker et al. 2018; Walpole et al. 2019) to investigate the M^pro^ binding of ligands initially identified by crystallographic screening, as well as molecules designed specifically as non-covalent inhibitors of this protease. Our goal was to provide orthogonal information on ligand binding to that which could be gained by enzymatic activity assays conducted in parallel by other groups. STD-NMR is a proven method for characterising the binding of small molecules to biological macromolecules, able to provide both quantitative affinity information and structural data on the proximity of ligand chemical groups to the protein. Here, we provide detailed documentation on the NMR protocols used to record these data and highlight the advantages, limitations and assumptions underpinning our approach. Our aim is to assist the comparison of M^pro^ STD-NMR data with other quantitative measurements, and facilitate the consideration of these data when designing future M^pro^ inhibitors.

## Materials and methods

### Protein production and purification

We created a SARS-CoV-2 M^pro^ genetic construct in pFLOAT vector (Rogala et al. 2015), encoding for the viral protease and an N-terminal His_6_-tag separated by a modified human rhinovirus (HRV) 3C protease recognition site, designed to reconstitute a native M^pro^ N-terminus upon HRV 3C cleavage. The M^pro^ construct was transformed into *Escherichia coli* strain Rosetta(DE3) (Novagen) and transformed clones were pre-cultured at 37 °C for 5 h in lysogeny broth supplemented with appropriate antibiotics. Starter cultures were used to inoculate Terrific Broth Autoinduction Media (Formedium) supplemented with 10% v/v glycerol and appropriate antibiotics. Cell cultures were grown at 37 °C for 5 h and then cooled to 18 °C for 12 h. For ^15^ N isotopically enriched protein production transformed *E. coli* clones were grown overnight at 37 °C in 200 mL M9 minimal media starter cultures supplemented with antibiotics and ^15^ N NH_4_Cl. These cultures were then used to inoculate 4–8 L of similarly supplemented M9 minimal media cultures. Cells were grown at 37 °C until OD_600_ of ~ 0.6, at which point protein expression was induced by addition of 0.25 mM isopropyl β-d-1-thiogalactopyranoside and was allowed to proceed for 12 h at 18 °C. Bacterial cells were harvested by centrifugation at 5000×*g* for 15 min.

Cell pellets were resuspended in 50 mM trisaminomethane (Tris)-Cl pH 8, 300 mM NaCl, 10 mM imidazole buffer, incubated with 0.05 mg/ml benzonase nuclease (Sigma Aldrich) and lysed by sonication on ice. Lysates were clarified by centrifugation at 50,000×*g* at 4 °C for 1 h. Lysate supernatants were loaded onto a HiTrap Talon metal affinity column (GE Healthcare) pre-equilibrated with lysis buffer. Column wash was performed with 50 mM Tris–Cl pH 8, 300 mM NaCl and 25 mM imidazole, followed by protein elution using the same buffer and an imidazole gradient from 25 to 500 mM concentration. The His_6_-tag was cleaved using home-made His_6_-tagged HRV 3C protease. The HRV 3C protease and the cleaved tag were removed by reverse metal affinity using a HiTrap Talon column. Flow-through fractions were concentrated and applied to a Superdex75 26/600 size exclusion column (GE Healthcare) equilibrated in NMR buffer (150 mM NaCl, 20 mM Na_2_HPO_4_ pH 7.4).

### Nuclear magnetic resonance (NMR) spectroscopy

All NMR experiments were performed using a 950 MHz solution-state instrument comprising an Oxford Instruments superconducting magnet, Bruker Avance III console and TCI probehead. A Bruker SampleJet sample changer was used for sample manipulation. Experiments were performed using TopSpin (Bruker). For direct STD-NMR measurements, samples comprised 10 μM M^pro^ and variable concentrations (20 μM–4 mM) of ligand compounds formulated in NMR buffer supplemented with 10% v/v D_2_O and deuterated dimethyl sulfoxide (*d*_6_-DMSO, 99.96% D, Sigma Aldrich) to 5% v/v final *d*_6_-DMSO concentration. In competition experiments, samples comprised 2 μM M^pro^, 0.8 mM of ligand  x0434 and variable concentrations (0–20 μM) of competing compound in NMR buffer supplemented with D_2_O and *d*_6_-DMSO as above. Sample volume was 140 μL and samples were loaded in 3 mm outer diameter SampleJet NMR tubes (Bruker) placed in 96-tube racks. NMR tubes were sealed with POM balls. For heteronuclear 2D spectra samples were formulated in 310 μL final volume in NMR buffer supplemented with D_2_O and *d*_6_-DMSO as above, 25 μM ^15^N-enriched M^pro^ unless otherwise indicated, and either 50 μM ligand or no ligand present. ^15^N M^pro^ samples were placed in 5 mm outer diameter advanced NMR microtubes (Shigemi) matched to D_2_O.

**Fig. 1:**
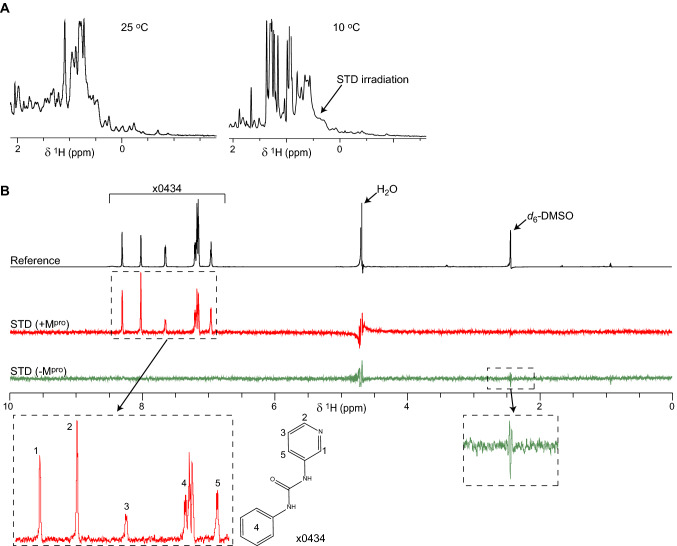
*1D and STD-NMR spectra of SARS-CoV-2 M*^*pro*^*.* **a** Methyl regions from ^1^H NMR spectra of recombinant SARS-CoV-2 M^pro^. The spectrum on the left was recorded from a 10 μM protein concentration sample in a 5 mm NMR tube at 25 °C using an excitation sculpting water-suppression method (Hwang and Shaka 1995). 512 acquisitions with recycle delay of 1.25 s were averaged, for a total experiment time of just over 10 min. The spectrum on the right was recorded from a 10 μM M^pro^ sample in a 3 mm NMR tube at 10 °C, using the same pulse sequence and acquisition parameters. For both spectra, data were processed with a quadratic sine function prior to Fourier transformation. Protein resonances are weaker in the 10 °C spectrum due to lower temperature and the reduced amount of sample used for acquisition in the smaller NMR tube. The position where on-resonance irradiation was applied for STD spectra is indicated. **b** Vertically offset ^1^H STD-NMR spectra from ligand  x0434 binding to M^pro^. The reference spectrum is in black with the  x0434, H_2_O and DMSO ^1^H resonances indicated. The STD spectrum of  x0434 in the presence of M^pro^ is shown in red while that in the absence of M^pro^ is in green. STD spectra are scaled up 64 × compared to the reference spectrum. Bottom panels correspond to magnified views of the indicated spectral regions, with  x0434 resonances assigned to chemical groups of that ligand as shown

STD-NMR experiments were performed at 10 °C using a pulse sequence described previously (Mayer and Meyer 1999) and an excitation sculpting water-suppression scheme (Hwang and Shaka 1995). Protein signals were suppressed in STD-NMR by the application of a 30 ms spin-lock pulse. We collected time-domain data of 16,384 complex points and 41.6 μsec dwell time (12.02 kHz sweepwidth). Data were collected in an interleaved pattern, with on- and off-resonance irradiation data separated into 16 blocks of 16 transients each (256 total transients per irradiation frequency). Transient recycle delay was 4 s and on- or off-resonance irradiation was performed using 0.1 mW of power for 3.5 s at 0.5 ppm or 26 ppm, respectively, for a total experiment time of approximately 50 min. Data were processed using TopSpin (Bruker). Reconstructed time-domain data from the difference of on- and off-resonance irradiation (STD spectra) or only the off-resonance irradiation (reference spectra) were processed by applying a 2 Hz exponential line broadening function and twofold zero-filling prior to Fourier transformation. Phasing parameters were derived for each sample from the reference spectra and copied to the STD spectra. ^1^H peak intensities were integrated in TopSpin using a local-baseline adjustment function. Data fitting to extract K_d_ values were performed in OriginPro (OriginLab). The folded state of M^pro^ in the presence of each ligand was verified by collecting ^1^H NMR spectra similar to Fig. 1a from all samples ahead of STD-NMR experiments.

Heteronuclear 2D ^1^H-^15^N spectra of M^pro^ were recorded at 25 °C using a SOFAST-HMQC pulse sequence from the biomolecular NMR group of the Institut de Biologie Structural in Grenoble (Favier and Brutscher 2019). We collected time-domain data of 756 complex points and 32.9 μsec dwell time (15.18 kHz sweepwidth) in the ^1^H dimension, and 64 complex points and 346.4 ms incrementation (2.89 kHz sweepwidth) in the indirect ^15^N dimension. Recycle delay was set to 0.2 s and 750 transients were collected per indirect increment, for a total experiment time of 7.4 h. Data were processed using NMRPipe (Delaglio et al. 1995) by applying a quadratic sine-function and tenfold zero filling along each spectra dimension prior to Fourier transformation. Spectra were visualised and overlaid in Sparky (Goddard et al. 2007).

### Ligand handling

Compounds for the initial STD-NMR assessment of crystallographic fragment binding to M^pro^ were provided by the XChem group at Diamond Light Source in the form of a 384-well plated library (DSI-poised, Enamine), with compounds dissolved in *d*_6_-DMSO at 500 mM nominal concentration. 1 μL of dissolved compounds was aspirated from this library and immediately mixed with 9 μL of *d*_6_-DMSO for a final fragment concentration of 50 mM, from which NMR samples were formulated. For titrations of the same crystallographic fragments compounds were procured directly from Enamine in the form of lyophilized powder, which was dissolved in *d*_6_-DMSO to derive compound stocks at 10 mM and 100 mM concentrations for NMR sample formulation.

STD-NMR assays of bespoke M^pro^ ligands used compounds commercially synthesised for COVID Moonshot. These ligands were provided to us by the XChem group in 96-well plates, containing 0.7 μL of 20 mM *d*_6_-DMSO-disolved compound per well. Plates were created using an Echo liquid handling robot (Labcyte) and immediately sealed and frozen at −20 °C. For use, ligand plates were thoroughly defrosted at room temperature and spun at 3500×*g* for 5 min. In single-concentration STD-NMR experiments, 140 μL of a pre-formulated mixture of M^pro^ and NMR buffer with D_2_O and *d*_6_-DMSO were added to each well to create the final NMR sample. For STD-NMR competition experiments, 0.5 μL of ligands were aspirated from the plates and immediately mixed with 19.5 μL of *d*_6_-DMSO for final ligand concentration of 0.5 mM from which NMR samples were formulated. For 2D heteronuclear NMR spectra ligands were provided by the XChem group in 96-well plates containing pre-aliquoted compounds as above, where the pre-formulated mixture of protein and buffer was added.

### Molecular dynamics (MD) simulations

The monomeric complexes of M^pro^ bound to chemical fragments were obtained from the RCSB Protein Data Bank entries 5R81 (ligand  x0195), 5REB ( x0387), 5RGI ( x0397), 5RGK ( x0426), 5R83 ( x0434) and 5REH ( x0540) for MD simulations with GROMACS version 2018 (Abraham et al. 2015) and the AMBER99SB-ILDN force field (Lindorff-Larsen et al. 2010). All complexes were inserted in a pre-equilibrated box containing water implemented using the TIP3P water model (Lindorff-Larsen et al. 2010). Force field parameters for the six ligands were generated using the general Amber force field and HF/6 – 31G*– derived RESP atomic charges (Bayly et al. 1993). The reference system consisted of the protein, the ligand, ~ 31,400 water molecules, 95 Na and 95 Cl ions in a 100 × 100 × 100 Å simulation box, resulting in a total number of ~ 98,000 atoms. Each system was energy-minimized and subsequently subjected to a 20 ns MD equilibration, with an isothermal-isobaric ensemble using isotropic pressure control (Bussi et al. 2009), and positional restraints on protein and ligand coordinates. The resulting equilibrated systems were replicated 4 times and independent 200 ns MD trajectories were produced with a time step of 2 fs, in constant temperature of 300 K, using separate v-rescale thermostats (Bussi et al. 2009) for the protein, ligand and solvent molecules. Lennard–Jones interactions were computed using a cut-off of 10 Å and electrostatic interactions were treated using particle mesh Ewald (Darden et al. 1993) with the same real-space cut-off. Analysis on the resulting trajectories was performed using MDAnalysis (Michaud-Agrawal et al. 2011; Gowers et al. 2016). Structures were visualised using PyMOL (DeLano 2002).

### Notes

The enzymatic inhibition potential of M^pro^ ligands, measured by RapidFire mass spectrometry (Achdout et al. 2020), was retrieved from the Collaborative Drug Discovery database (CDD database 2021).

## Results

### STD-NMR assays of M^pro^ ligand binding

M^pro^ forms dimers in crystals via an extensive interaction interface involving two domains (Zhang et al. 2020). M^pro^ dimers likely have a sub-μM solution dissociation constant (K_d_) by analogy to previously studied 3C-like coronavirus proteases (Grum-Tokars et al. 2008). At the 10 μM protein concentration of our NMR assays M^pro^ is, thus, expected to be dimeric with an estimated molecular weight of nearly 70 kDa. Despite the relatively large size of M^pro^ for solution NMR, ^1^H spectra of the protease readily showed the presence of multiple up-field shifted (< 0.5 ppm) peaks corresponding to protein methyl groups (Fig. 1a). In addition to demonstrating that M^pro^ is folded under the conditions tested, these spectra allowed us to identify the chemical shifts of M^pro^ methyl groups that may be suitable for on-resonance irradiation in STD-NMR experiments. Trials with on-resonance irradiation applied to different methyl group peaks showed that irradiating at 0.5 ppm (Fig. 1a) produced the strongest STD signal from ligands in the presence of M^pro^, while simultaneously avoiding ligand excitation that would yield false-positive signals in the absence of M^pro^ (Fig. 1b). Further, we noted that small molecules abundant in the samples but not binding specifically to M^pro^, such as DMSO, produced pseudo-dispersive residual signal lineshapes in STD spectra, while true M^pro^ ligands produced peaks in STD with absorptive ^1^H lineshapes. We surmised that STD-NMR is suitable for screening ligand binding to M^pro^, requiring relatively small amounts (10–50 μgr) of protein and time (under 1 h) per sample studied.

The strength of STD signal is quantified by calculating the ratio of integrated signal intensity of peaks in the STD spectrum over that of the reference spectrum (STD_ratio_). The STD_ratio_ factor is inversely proportional to ligand K_d_, as ${STD}_{ratio}\propto \frac{1}{{K}_{d}+[L]}$ where [L] is ligand concentration. Measuring STD_ratio_ values over a range of ligand concentrations allows fitting of the proportionality constant and calculation of ligand K_d_. However, time and sample-amount considerations, including the limited availability of bespoke compounds synthesized for the COVID Moonshot project, made recording full STD-NMR titrations impractical for screening hundreds of ligands. Thus, we evaluated whether measuring the STD_ratio_ value at a single ligand concentration may be an informative alternative to K_d_, provided restraints could be placed, for example, on the proportionality constant.

Theoretical and practical considerations suggested that three parameters influence our evaluation of single-concentration STD_ratio_ values towards an affinity context. Firstly, the STD_ratio_ factor is affected by the efficiency of NOE magnetisation transfer between protein and ligand, which in turn depends on the proximity of ligand and protein groups, and the chemical nature of these groups (Mayer and Meyer 1999; Becker et al. 2018; Walpole et al. 2019). To minimize the influence of these factors across diverse ligands, we sought to quantify the STD_ratio_ of only aromatic ligand groups, and only consider those showing the strongest STD signal; thus, that are in closest proximity to the protein. Second, STD-NMR assays require ligand exchange between protein-bound and -free states in the timeframe of the experiment; strongly bound compounds that dissociate very slowly from the protein would yield reduced STD_ratio_ values compared to weaker ligands that dissociate more readily. Structures of M^pro^ with many different ligands show that the protein conformation does not change upon complex formation and that the active site is fully solvent-exposed (Douangamath et al. 2020), which suggests that ligand association can proceed with high rate (10^7^–10^8^ M^−1^ s^−1^). Under this assumption, the ligand dissociation rate is the primary determinant of interaction strength. Given the duration of the STD-NMR experiment in our assays, and the ratios of ligand:protein used, we estimated that significant protein—ligand exchange will take place even for interactions as strong as low-mM K_d_. Finally, uncertainties or errors in nominal ligand concentration skew the correlation of STD_ratio_ to compound affinities; as shown in Fig. S1, STD_ratio_ values increase strongly when very small amounts of ligands are assessed. Thus, overly large STD_ratio_ values may be measured if ligand concentrations are significantly lower than anticipated.

### Quantitating M^pro^ binding of ligands identified by crystallographic screening

Mindful of the limitations inherent to measuring single-concentration STD_ratio_ values, and prior to using STD-NMR to evaluate bespoke M^pro^ ligands, we used this method to assess binding to the protease of small chemical fragments identified in crystallographic screening experiments (Douangamath et al. 2020). In crystallographic screening campaigns of other target proteins such fragments were seen to have very weak affinities (> 1 mM K_d_, e.g. Davies et al. 2016), thereby satisfying the exchange criterion set out above. 39 non-covalent M^pro^ interactors are part of the DSI-poised fragment library to which we were given access, comprising 17 active site binders, two compounds targeting the M^pro^ dimerisation interface and 20 molecules binding elsewhere on the protein surface (Douangamath et al. 2020). We initially recorded STD-NMR spectra from these compounds in the absence of M^pro^ to confirm that we obtained no or minimal STD signal when protease is omitted, and to verify ligand identity from reference ^1^H spectra. Five ligands gave no solution NMR signal or produced reference ^1^H spectra inconsistent with the compound chemical structure; these ligands were not evaluated further. Samples of 10 μM M^pro^ and 0.8 mM nominal ligand concentration were then formulated from the remaining 34 compounds (Table S1), and STD-NMR spectra were recorded, from which only aromatic ligand STD signals were considered for further analysis.

**Fig. 2:**
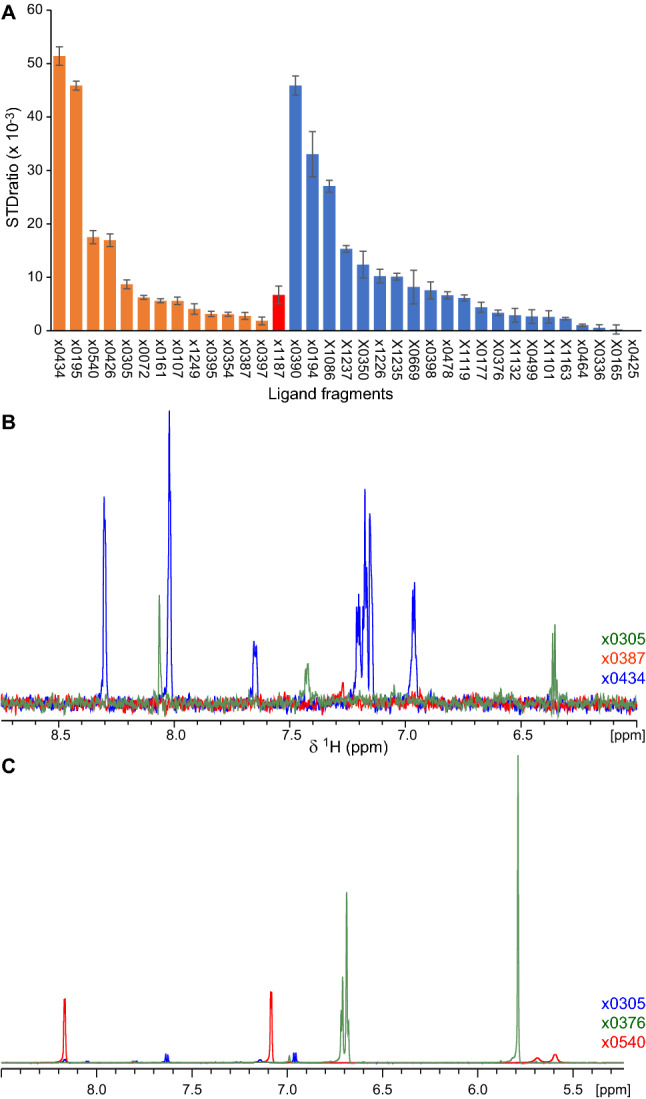
Assessment of fragment binding to M^pro^. **a** STD_ratio_ values for chemical fragments identified by crystallographic screening as binding to M^pro^ (Douangamath et al. 2020). Ligands binding to the M^pro^ active site are coloured orange, at the M^pro^ dimer interface in red, and elsewhere on the protein surface in blue. **b** Overlay of STD-NMR spectra from fragments  x0305,  x0387 and  x0434, which bind the M^pro^ active site, showing the ligand aromatic region in the presence of M^pro^. Spectra are colour coded per ligand as indicated. As seen, the three fragments yield significantly different STD signal intensities captured in the STD_ratio_ values shown in (**a**). **c** Overlay of reference spectra from fragments  x0305,  x0376 and  x0540, showing the ligand aromatic region. Peak intensities vary substantially, suggesting significant differences in ligand concentration

We observed large variations in STD signal intensity and STD_ratio_ values in the presence of M^pro^ across compounds (Fig. 2a, b; Table S1), with many ligands producing little or no STD signal, suggesting substantial differences in compound affinity for the protease. However, we also noted that ligand reference spectra differed substantially in intensity (Fig. 2c), despite compounds being at the same nominal concentration. Integrating ligand peaks in these reference spectra revealed differences in per-^1^H intensity of up to ~ 15-fold (Table S1). Such differences in ligand signal may arise from parameters of the NMR experiment, such as sample centering and calibration, from errors in sample formulation, or alternatively from concentration inconsistencies in the compound library and ligand aggregation in solution. To evaluate these possibilities we integrated the residual ^1^H signal of *d*_6_-DMSO in our reference spectra, which acts as internal control being sensitive to the same NMR parameters and formulation errors as the ligands. We found that DMSO signal varied by less than 35% across any pair of samples (11% average deviation). Thus, we concluded that NMR parameters and sample formulation errors may have contributed differences in ligand signal of up to ~ 1/3, but did not account for the ~ 15-fold signal differences observed. This suggests that effective ligand concentrations in solution vary substantially.

Given that differences in effective compound concentration can skew the relative STD_ratio_ values of ligands (Fig. S1), and that such concentration differences were also observed among newly designed M^pro^ inhibitors (see below), we questioned whether recording STD_ratio_ values under these conditions can provide useful information. To address this question we attempted to quantify the affinity of crystallographic fragments to M^pro^, selecting ligands that showed clear differences in STD_ratio_ values in the assays above and focusing on compounds binding at the M^pro^ active site; hence, that are of potential interest to inhibitor development. We performed M^pro^ binding titrations monitored by STD-NMR of compounds  x0195,  x0354,  x0426 and  x0434 in 50 μM–4 mM concentrations (Fig. S2), and noted that only compounds  x0434 and  x0195, which show the highest STD_ratio_ (Fig. 2a), bound strongly enough for an affinity constant to be estimated (K_d_ of 1.6 ± 0.2 mM and 1.7 ± 0.2 mM, respectively). In contrast, the titrations of x0354 and  x0426, which yielded lower STD_ratio_ values, could not be fit to extract a K_d_ indicating weaker binding to M^pro^.

To further this analysis, we assessed the binding of fragments  x0195, x0387,  x0397,  x0426,  x0434 and  x0540 to the M^pro^ active site using atomistic molecular dynamics (MD) simulations of 200 nsec duration. As shown in Fig. S3a, b, and Movies S1 and S2, fragments with high STD_radio_ values ( x0434 and  x0195) always located in the M^pro^ active site despite exchanging between different binding conformations (Fig. S4), with average ligand root-mean-square-deviation (RMSD) of 3.2 Å and 5.1 Å respectively after the first 100 nsec of simulation. Medium STD_ratio_ value fragments ( x0426 and  x0540, Fig. S3c, d, and Movies S3 and S4) show average RMSDs of approximately 9 Å in the same simulation timeframe, frequently exchanging to alternative binding poses and with  x0540 occasionally exiting the M^pro^ active site. In contrast, fragments showing very little STD NMR signal ( x0397 and  x0387, Fig. S3e, f, and Movies S5 and S6) regularly exit the M^pro^ active site and show average RMSDs in excess of 15 Å with very limited stability. Combining the quantitative K_d_ and MD information above, we surmised that, despite limitations inherent in this type of analysis and uncertainties in ligand amounts in solution, STD_ratio_ values recorded at single compound concentration can act as proxy measurements of M^pro^ affinity for ligands.

### Assessment of M^pro^ binding by COVID Moonshot ligands

**Fig. 3:**
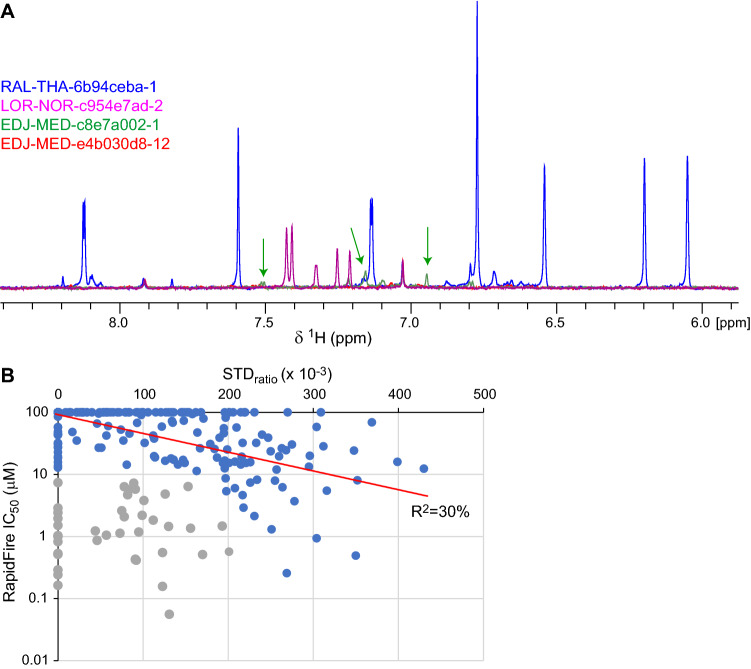
STD-NMR of COVID Moonshot ligands binding to M^pro^. **a** Overlay of reference spectra from the indicated COVID Moonshot ligands, showing the ligand aromatic region in each case. Spectra are colour coded per ligand as indicated. As seen, peak intensities vary substantially, suggesting significant differences in ligand concentration. Peaks of ligand EDJ-MED-c8e7a002-1 (green) are indicated by arrows; ligand EDJ-MED-e4b030d8-12 (red) produced no peaks in the NMR spectrum. **b** Plot of STD_ratio_ values from COVID Moonshot ligands assessed by STD-NMR against their IC_50_ value estimated by RapidFire mass spectrometry enzymatic assays (Achdout et al. 2020). Ligands in blue show weak correlation between the two methods (red line, corresponding to an exponential function along the IC_50_ dimension). Ligands in grey represent outliers of the STD-NMR or enzymatic method as discussed

We proceeded to characterise by STD-NMR the M^pro^ binding of bespoke ligands created as part of the COVID Moonshot project and designed to act as non-covalent inhibitors of the protease (Achdout et al. 2020). Similar to the assays of crystallographic fragments above, we focused our analysis of STD signals to aromatic moieties of ligands binding to the M^pro^ active side and extracted STD_ratio_ values only from the strongest STD peaks. Once again, we noted substantial differences in effective compound concentrations, judging from reference ^1^H spectral intensities (Fig. 3a). These differences, which may reflect ligand aggregation, could not be attributed to errors in NMR parameters or sample preparation as the standard deviation of residual ^1^H intensity in the *d*_6_-DMSO peak did not exceed 5% in any of the ligand batches tested. Crucially, out of 650 different molecules tested, samples of 35 compounds (7.6%) yielded no detectable NMR signal and 86 (13.2%) very little signal (Fig. 3a). In these cases, NMR assays were repeated using a separate batch of compound; however, 96.2% of repeat experiments yielded the same outcome of no or very little NMR signal from the ligands.

We measured STD_ratio_ values from samples where ligands produced sufficiently strong reference ^1^H NMR spectra to be readily visible, and deposited these values and associated raw NMR data to the Collaborative Drug Discovery database (CDD database 2021). Some of these ligands were assessed independently for enzymatic inhibition of M^pro^ using a mass spectrometry method as part of the COVID Moonshot collaboration (Achdout et al. 2020). Where both parameters are available, we compared the STD_ratio_ values and 50% inhibition concentrations (IC_50_) of these ligands. As shown in Fig. 3b, STD_ratio_ and IC_50_ values show weak correlation (R^2^ = 30%) for most ligands tested; however, a subset of ligands displayed conspicuously low or even no STD signals considering their effect on M^pro^ activity, and presented themselves as outliers in the correlation graph. As these outlier ligands had IC_50_ values below 10 μM, suggesting that their affinities to the protease may be in the μM K_d_ region, we considered whether our approach gives rise to false-negative STD results, for example through slow ligand dissociation from M^pro^.

**Fig. 4:**
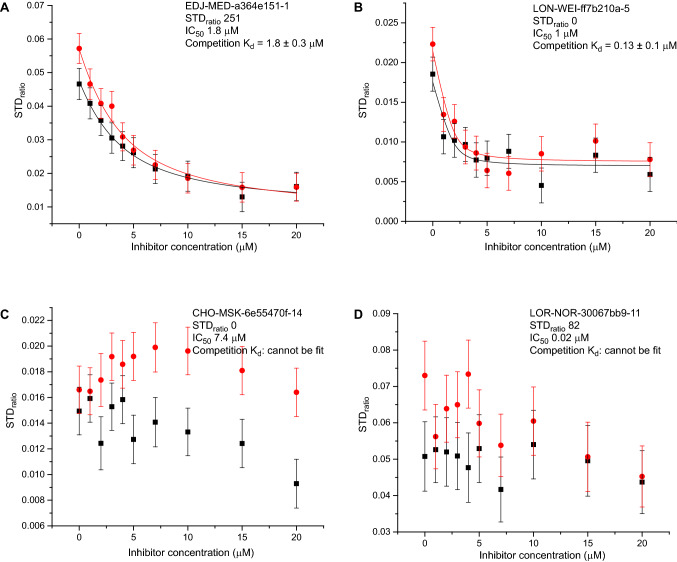
*Quantification of interaction affinities for COVID Moonshot ligands binding to M*^*pro*^. Shown here are plots of STD_ratio_ values derived from two NMR peaks of fragment  x0434 as function of a competing COVID Moonshot ligand concentration. For each ligand, the STD_ratio_ values derived from single-point assays and the IC_50_ values from RapidFire enzymatic assays are indicated. The titrations of COVID Moonshot ligands EDJ-MED-a364e151-1 (**a**) and LON-WEI-ff7b210a-5 (**b**) could be fit to an ideal competition model to derive binding affinities for these compounds; in contrast, ligands CHO-MSK-6e55470f-14 (**c**) and LOR-NOR-30067bb9-11 (**d**) failed to displace fragment  x0434 from the M^pro^ active site in a significant manner

To address this question, we derived an assay whereby the bespoke, high-affinity M^pro^ inhibitor would outcompete a lower-affinity ligand known to provide strong STD signal from the protease active site. In these experiments the lower-affinity ligand would act as ‘spy’ molecule whose STD signal reduces as function of inhibitor concentration. We used fragment  x0434, which yields substantial STD signal with M^pro^ (Figs. 1b and 2a), as ‘spy’, and tested protease inhibitors EDJ-MED-a364e151-1, LON-WEI-ff7b210a-5, CHO-MSK-6e55470f-14 and LOR-NOR-30067bb9-11 as  x0434 competitors. Of these inhibitors, EDJ-MED-a364e151-1 gave rise to substantial STD signal in earlier assays, whereas the remaining produced little or no STD signal; yet, all four inhibitors were reported to have low-µM or sub-µM IC_50_ values based on M^pro^ enzymatic assays. In these competition experiments, both EDJ-MED-a364e151-1 and LON-WEI-ff7b210a-5 yielded K_d_ parameters comparable to the reported IC_50_ values (Fig. 4a, b), showing that at least in the case of LON-WEI-ff7b210a-5 the absence of STD signal in the single-concentration NMR assays above represented a false-negative result. In contrast, CHO-MSK-6e55470f-14 and LOR-NOR-30067bb9-11 were unable to compete x0434 from the protease active site (Fig. 4c, d), suggesting that in these two cases the reported IC_50_ values do not reflect inhibitor binding to the protease, and that the weak STD signal of the initial assays was a better proxy of affinity. We surmised that although some low STD_ratio_ values of M^pro^ inhibitors may not accurately reflect compound affinity to the protease, such values cannot be discounted as a whole as they may correspond to non-binding ligands.

To further this analysis, we attempted to evaluate binding of the same protease inhibitors to M^pro^ using protein-observed heteronuclear 2D spectra. In such spectra protein resonances are expected to shift as a result of compound binding due to alterations in the chemical environment of the binding site caused by the ligand. We produced ^15^N isotopically enriched M^pro^ and recorded spectra using a number of different NMR experiments optimised for rapid data acquisition and signal accumulation, including SOFAST-HMQC, BEST-HSQC and BEST-TROSY (Favier and Brutscher 2019). We obtained the best M^pro^ spectra using the SOFAST-HMQC pulse sequence; however, we noted that spectral quality, including signal-to-noise obtained per unit time, was reduced as function of protein concentration, which is indicative of M^pro^ aggregation under the conditions used (Fig. S5a, b). As a result, we proceeded to record spectra at 25 μM M^pro^ concentration, which necessitated almost 7.5 h of acquisition time per spectrum to accumulate adequate signal-to-noise.

Despite these efforts, the resulting SOFAST-HMQC spectra of M^pro^ were of relatively low resolution and signal quality (Fig. S5b), and displayed in the order of tens of discrete peaks (for reference, we would expect one peak per amino acid residue except for prolines; hence, more than 290 discrete peaks for M^pro^). Addition of ligands in concentrations that should result in M^pro^ saturation based on reported ligand IC_50_ values yielded no changes to the vast majority of peaks in the NMR spectra, with only a small number of resonances showing slight perturbations (Fig. S5c, d). As assignments of M^pro^ NMR resonances have not yet been reported, we were unable to confirm whether the slight perturbations observed upon ligand addition corresponded to binding events at the protease active site, or alternatively non-specific interactions elsewhere on the protein. Given the difficulty in obtaining these 2D spectra, and the ambiguous nature of their interpretation, we concluded that such protein-observed experiments are not a suitable NMR method for characterising ligand binding to M^pro^.

## Discussion

Fragment-based screening is a tried and tested method for reducing the number of compounds that need to be assessed for binding against a specific target in order to sample chemical space (Erlanson et al. 2016). Combined with X-ray crystallography, which provides information on the target site and binding pose of ligands, initial fragments can quickly be iterated into potent and specifically-interacting compounds. The COVID Moonshot collaboration (Achdout et al. 2020) took advantage of crystallographic fragment-based screening (Douangamath et al. 2020) to initiate the design of novel inhibitors targeting the essential main protease of the SARS-CoV-2 coronavirus; however crystallographic structures do not report on ligand affinity and inhibitory potency in enzymatic assays does not always correlate with ligand binding. Thus, supplementing these methods with solution NMR tools highly sensitive to ligand binding can provide a powerful combination of orthogonal information and assurance against false starts. Our primary aim in the study presented here was to provide exactly this type of information for COVID Moonshot. However, we recognise that many of the problems encountered in this work are likely to reoccur in the context of other ligand screening efforts; hence, we hope that this report will prove informative to a more general audience.

A key issue encountered early in our effort to characterise M^pro^-interacting ligands was compound quality. An initial assessment of M^pro^ ligands drawn from a commercial library showed large variation of up to 15-fold in effective ligand concentration (Fig. 2c). Ligand quality control is a recognised problem in screening campaigns stemming from a multitude of factors, including the exact amount of compounds provided by vendors, storage conditions, and ligand degradation and aggregation in aqueous buffers, among others (Lepre 2011). A recent study aiming to develop a robust fragment library for high-throughput screening reported that up to approximately 30% of fragments failed to pass one or more quality-control metrics, including ligand concentration (Sreeramulu et al. 2020). Our own experience with bespoke M^pro^ ligands was that ~ 20% of compounds had low or very low effective concentration in solution (Fig. 3a). Although in the context of the COVID Moonshot project this proportion of poorly-behaved ligands could be tolerated in the interest of rapid progression, it is important to underline the effect of library quality in screening as this can give rise to both false-negative and false-positive results.

Despite these deficiencies, we showed that STD-NMR is a suitable method for characterising ligand binding to M^pro^, allowing us to assess ligand interactions using relatively small amounts of protein and in under 1 h of experiment time per ligand (Fig. 1b). However, screening compounds in a high-throughput manner is not compatible with the time- and ligand-amount requirements of full STD-NMR titrations. Thus, we resorted to using an unconventional metric, the single-concentration STD_ratio_ value, as proxy for ligand affinity. Although this metric has limitations due to its dependency on magnetisation transfer between protein and ligand, and on relatively rapid exchange between the ligand-free and -bound states, we demonstrated that it can nevertheless be informative. Specifically, the relative STD_ratio_ values of chemical fragments bound to the M^pro^ active site provided insight on fragment affinity (Fig. 2a), as crosschecked by quantitative titrations (Fig. S2) and MD simulations (Fig. S3). Furthermore, STD_ratio_ values of COVID Moonshot compounds held a weak correlation to enzymatic IC_50_ parameters (Fig. 3b), although false-negative and -positive results from both methods contribute to multiple outliers. Thus, in our view the biggest limitation of using the single-concentration STD_ratio_ value as metric relates to its supra-linear sensitivity to effective ligand concentration (Fig. S1), which can vary substantially across ligands in a large project (Fig. 3a).

How then should the STD data recorded as part of COVID Moonshot be used? Firstly, we showed that at least for some bespoke M^pro^ ligands the STD_ratio_ value obtained is a better proxy for compound affinity compared to IC_50_ parameters from enzymatic assays (Fig. 4). This, inherently, is the value of employing orthogonal methods thereby minimizing the number of potential false results. Thus, when one is considering existing M^pro^ ligands to base the design of future inhibitors, a high STD_ratio_ value as well as low IC_50_ parameters are both desirable. Second, due to the aforementioned limitations of single-concentration STD_ratio_ value as proxy of affinity, and the influence of uncertainties in ligand concentrations, we believe that comparisons of compounds and derivatives differing by less than ~ 50% in STD_ratio_ is not meaningful. Rather, we propose that the STD_ratio_ values of M^pro^ ligands measured and available at the CDD database should be treated as a qualitative metrics of compound affinity.

In conclusion, we presented here protocols for the assessment of SARS-CoV-2 M^pro^ ligands using STD-NMR spectroscopy, and evaluated the relative qualitative affinities of chemical fragments and compounds designed as part of COVID Moonshot. Although development of novel antivirals to combat COVID-19 is still at an early stage, we hope that this information will prove valuable to groups working towards such treatments.

## Supplementary Information

Below is the link to the electronic supplementary material.

Supplementary figures (PDF 5271 kb)

Supplementary movie 1 (MP4 9541 kb)

Supplementary movie 2 (MP4 9548 kb)

Supplementary movie 3 (MP4 9550 kb)

Supplementary movie 4 (MP4 9540 kb)

Supplementary movie 5 (MP4 9546 kb)

Supplementary movie 6 (MP4 9533 kb)

Supplementary material 7 (DOCX 134 KB)

## Data Availability

Raw STD-NMR data and extracted STD_ratio_ parameters have been deposited in the Collaborative Drug Discovery database and are publicly available with free registration. All other data and materials are available on request.

## References

[CR1] Abraham MJ, Murtola T, Schulz R, Páll S, Smith JC, Hess B, Lindahl E (2015). GROMACS: high performance molecular simulations through multi-level parallelism from laptops to supercomputers. SoftwareX.

[CR2] Achdout H, Aimon A, Bar-David E, Barr H, Ben-Shmuel A, Bennett J, Bobby ML, Brun J, Sarma B, Calmiano M, Carbery A, Cattermole E, Chodera JD, Clyde A, Coffland JE, Cohen G, Cole J, Contini A, Cox L, Cvitkovic M, Dias A, Douangamath A, Duberstein S, Dudgeon T, Dunnett L, Eastman PK, Erez N, Fairhead M, Fearon D, Fedorov O, Ferla M, Foster H, Foster R, Gabizon R, Gehrtz P, Gileadi C, Giroud C, Glass WG, Glen R, Glinert I, Gorichko M, Gorrie-Stone T, Griffen EJ, Heer J, Hill M, Horrell S, Hurley MFD, Israely T, Jajack A, Jnoff E, John T, Kantsadi AL, Kenny PW, Kiappes JL, Koekemoer L, Kovar B, Krojer T, Lee AA, Lefker BA, Levy H, London N, Lukacik P, Macdonald HB, MacLean B, Malla TR, Matviiuk T, McCorkindale W, Melamed S, Michurin O, Mikolajek H, Morris A, Morris GM, Morwitzer MJ, Moustakas D, Neto JB, Oleinikovas V, Overheul GJ, Owen D, Pai R, Pan J, Paran N, Perry B, Pingle M, Pinjari J, Politi B, Powell A, Psenak V, Puni R, Rangel VL, Reddi RN, Reid SP, Resnick E, Robinson MC, Robinson RP, Rufa D, Schofield C, Shaikh A, Shi J, Shurrush K, Sittner A, Skyner R, Smalley A, Smilova MD, Spencer J, Strain-Damerell C, Swamy V, Tamir H, Tennant R, Thompson A, Thompson W, Tomasio S, Tumber A, Vakonakis I, van Rij RP, Varghese FS, Vaschetto M, Vitner EB, Voelz V, von Delft A, von Delft F, Walsh M, Ward W, Weatherall C, Weiss S, Wild CF, Wittmann M, Wright N, Yahalom-Ronen Y, Zaidmann D, Zidane H, Zitzmann N (2020). COVID moonshot: open science discovery of SARS-CoV-2 main protease inhibitors by combining crowdsourcing, high-throughput experiments, computational simulations, and machine learning. bioRxiv.

[CR3] Bayly CI, Cieplak P, Cornell W, Kollman PA (1993). A well-behaved electrostatic potential based method using charge restraints for deriving atomic charges: the RESP model. J Phys Chem.

[CR4] Becker W, Bhattiprolu KC, Gubensak N, Zangger K (2018). Investigating protein-ligand interactions by solution nuclear magnetic resonance spectroscopy. ChemPhysChem.

[CR5] Bermingham A, Chand MA, Brown CS, Aarons E, Tong C, Langrish C, Hoschler K, Brown K, Galiano M, Myers R, Pebody RG, Green HK, Boddington NL, Gopal R, Price N, Newsholme W, Drosten C, Fouchier RA, Zambon M (2012). Severe respiratory illness caused by a novel coronavirus, in a patient transferred to the United Kingdom from the Middle East, September 2012. Euro Surveill.

[CR6] Bredenbeek PJ, Pachuk CJ, Noten AF, Charite J, Luytjes W, Weiss SR, Spaan WJ (1990). The primary structure and expression of the second open reading frame of the polymerase gene of the coronavirus MHV-A59; a highly conserved polymerase is expressed by an efficient ribosomal frameshifting mechanism. Nucleic Acids Res.

[CR7] Bussi G, Zykova-Timan T, Parrinello M (2009). Isothermal-isobaric molecular dynamics using stochastic velocity rescaling. J Chem Phys.

[CR8] Collaborative Drug Discovery database public access (2021). https://www.collaborativedrug.com/public-access/

[CR9] Darden T, York D, Pedersen L (1993). Particle mesh Ewald: an N⋅ log (N) method for Ewald sums in large systems. J Chem Phys.

[CR10] Davies TG, Wixted WE, Coyle JE, Griffiths-Jones C, Hearn K, McMenamin R, Norton D, Rich SJ, Richardson C, Saxty G, Willems HM, Woolford AJ, Cottom JE, Kou JP, Yonchuk JG, Feldser HG, Sanchez Y, Foley JP, Bolognese BJ, Logan G, Podolin PL, Yan H, Callahan JF, Heightman TD, Kerns JK (2016). Monoacidic inhibitors of the Kelch-like ECH-associated protein 1: nuclear factor erythroid 2-related factor 2 (KEAP1:NRF2) protein-protein interaction with high cell potency identified by fragment-based discovery. J Med Chem.

[CR11] Delaglio F, Grzesiek S, Vuister GW, Zhu G, Pfeifer J, Bax A (1995). NMRPipe: a multidimensional spectral processing system based on UNIX pipes. J Biomol NMR.

[CR12] DeLano WL (2002). The PyMOL molecular graphics system.

[CR13] Douangamath A, Fearon D, Gehrtz P, Krojer T, Lukacik P, Owen CD, Resnick E, Strain-Damerell C, Aimon A, Abranyi-Balogh P, Brandao-Neto J, Carbery A, Davison G, Dias A, Downes TD, Dunnett L, Fairhead M, Firth JD, Jones SP, Keeley A, Keseru GM, Klein HF, Martin MP, Noble MEM, O'Brien P, Powell A, Reddi RN, Skyner R, Snee M, Waring MJ, Wild C, London N, von Delft F, Walsh MA (2020). Crystallographic and electrophilic fragment screening of the SARS-CoV-2 main protease. Nat Commun.

[CR14] El-Baba TJ, Lutomski CA, Kantsadi AL, Malla TR, John T, Mikhailov V, Bolla JR, Schofield CJ, Zitzmann N, Vakonakis I, Robinson CV (2020). Allosteric inhibition of the SARS-CoV-2 main protease: insights from mass spectrometry based assays. Angew Chem Int Ed.

[CR15] Erlanson DA, Fesik SW, Hubbard RE, Jahnke W, Jhoti H (2016). Twenty years on: the impact of fragments on drug discovery. Nat Rev Drug Discov.

[CR16] Favier A, Brutscher B (2019). NMRlib: user-friendly pulse sequence tools for Bruker NMR spectrometers. J Biomol NMR.

[CR17] Ghosh AK, Xi K, Grum-Tokars V, Xu X, Ratia K, Fu W, Houser KV, Baker SC, Johnson ME, Mesecar AD (2007). Structure-based design, synthesis, and biological evaluation of peptidomimetic SARS-CoV 3CLpro inhibitors. Bioorg Med Chem Lett.

[CR18] Goddard TD, Kneller DG. SPARKY 3. University of California, San Francisco

[CR19] Gowers RJ, Linke M, Barnoud J, Reddy TJE, Melo MN, Seyler SL, Dotson DL, Domanski J, Buchoux S, Kenney M, Beckstein O (2016) MDAnalysis: a python package for the rapid analysis of molecular dynamics simulations. In: Benthall S (eds) 15th Python in Science Conference. Austin, TX, pp 98–105

[CR20] Grum-Tokars V, Ratia K, Begaye A, Baker SC, Mesecar AD (2008). Evaluating the 3C-like protease activity of SARS-Coronavirus: recommendations for standardized assays for drug discovery. Virus Res.

[CR21] Hilgenfeld R (2014). From SARS to MERS: crystallographic studies on coronaviral proteases enable antiviral drug design. FEBS J.

[CR22] Hwang TL, Shaka AJ (1995). Water suppression that works—excitation sculpting using arbitrary wave-forms and pulsed-field gradients. J Magn Reson Ser A.

[CR23] Kucharski AJ, Russell TW, Diamond C, Liu Y, Edmunds J, Funk S, Eggo RM, Centre for Mathematical Modelling of Infectious Diseases, C.-w.g (2020). Early dynamics of transmission and control of COVID-19: a mathematical modelling study. Lancet Infect Dis.

[CR24] Kuiken T, Fouchier RA, Schutten M, Rimmelzwaan GF, van Amerongen G, van Riel D, Laman JD, de Jong T, van Doornum G, Lim W, Ling AE, Chan PK, Tam JS, Zambon MC, Gopal R, Drosten C, van der Werf S, Escriou N, Manuguerra JC, Stohr K, Peiris JS, Osterhaus AD (2003). Newly discovered coronavirus as the primary cause of severe acute respiratory syndrome. Lancet.

[CR25] Lepre CA (2011). Practical aspects of NMR-based fragment screening. Methods Enzymol.

[CR26] Lindorff-Larsen K, Piana S, Palmo K, Maragakis P, Klepeis JL, Dror RO, Shaw DE (2010). Improved side-chain torsion potentials for the Amber ff99SB protein force field. Proteins.

[CR27] Mayer M, Meyer B (1999). Characterization of ligand binding by saturation transfer difference NMR spectroscopy. Angew Chem Int Ed Engl.

[CR28] Michaud-Agrawal N, Denning EJ, Woolf TB, Beckstein O (2011). MDAnalysis: a toolkit for the analysis of molecular dynamics simulations. J Comput Chem.

[CR29] Rogala KB, Dynes NJ, Hatzopoulos GN, Yan J, Pong SK, Robinson CV, Deane CM, Gonczy P, Vakonakis I (2015). The Caenorhabditis elegans protein SAS-5 forms large oligomeric assemblies critical for centriole formation. Elife.

[CR30] Rut W, Groborz K, Zhang L, Sun X, Zmudzinski M, Pawlik B, Wang X, Jochmans D, Neyts J, Mlynarski W, Hilgenfeld R, Drag M (2020). SARS-CoV-2 M(pro) inhibitors and activity-based probes for patient-sample imaging. Nat Chem Biol.

[CR31] Sreeramulu S, Richter C, Kuehn T, Azzaoui K, Blommers MJJ, Del Conte R, Fragai M, Trieloff N, Schmieder P, Nazare M, Specker E, Ivanov V, Oschkinat H, Banci L, Schwalbe H (2020). NMR quality control of fragment libraries for screening. J Biomol NMR.

[CR32] Thiel V, Ivanov KA, Putics A, Hertzig T, Schelle B, Bayer S, Weissbrich B, Snijder EJ, Rabenau H, Doerr HW, Gorbalenya AE, Ziebuhr J (2003). Mechanisms and enzymes involved in SARS coronavirus genome expression. J Gen Virol.

[CR33] Verschueren KH, Pumpor K, Anemuller S, Chen S, Mesters JR, Hilgenfeld R (2008). A structural view of the inactivation of the SARS coronavirus main proteinase by benzotriazole esters. Chem Biol.

[CR34] Walpole S, Monaco S, Nepravishta R, Angulo J (2019). STD NMR as a technique for ligand screening and structural studies. Methods Enzymol.

[CR35] WHO (2021) Coronavirus disease. https://www.who.int/emergencies/diseases/novel-coronavirus-2019

[CR36] Wu F, Zhao S, Yu B, Chen YM, Wang W, Song ZG, Hu Y, Tao ZW, Tian JH, Pei YY, Yuan ML, Zhang YL, Dai FH, Liu Y, Wang QM, Zheng JJ, Xu L, Holmes EC, Zhang YZ (2020). A new coronavirus associated with human respiratory disease in China. Nature.

[CR37] Yang H, Yang M, Ding Y, Liu Y, Lou Z, Zhou Z, Sun L, Mo L, Ye S, Pang H, Gao GF, Anand K, Bartlam M, Hilgenfeld R, Rao Z (2003). The crystal structures of severe acute respiratory syndrome virus main protease and its complex with an inhibitor. Proc Natl Acad Sci USA.

[CR38] Yang H, Xie W, Xue X, Yang K, Ma J, Liang W, Zhao Q, Zhou Z, Pei D, Ziebuhr J, Hilgenfeld R, Yuen KY, Wong L, Gao G, Chen S, Chen Z, Ma D, Bartlam M, Rao Z (2005). Design of wide-spectrum inhibitors targeting coronavirus main proteases. PLoS Biol.

[CR39] Zaki AM, van Boheemen S, Bestebroer TM, Osterhaus AD, Fouchier RA (2012). Isolation of a novel coronavirus from a man with pneumonia in Saudi Arabia. N Engl J Med.

[CR40] Zhang L, Lin D, Sun X, Curth U, Drosten C, Sauerhering L, Becker S, Rox K, Hilgenfeld R (2020). Crystal structure of SARS-CoV-2 main protease provides a basis for design of improved alpha-ketoamide inhibitors. Science.

[CR41] Zhu N, Zhang D, Wang W, Li X, Yang B, Song J, Zhao X, Huang B, Shi W, Lu R, Niu P, Zhan F, Ma X, Wang D, Xu W, Wu G, Gao GF, Tan W, China Novel Coronavirus, I. & Research, T (2020). A novel coronavirus from patients with pneumonia in China, 2019. N Engl J Med.

