# Supplementary material for: A COVID moonshot: assessment of ligand binding to the SARS-CoV-2 main protease by saturation transfer difference NMR spectroscopy
Source: J Biomol NMR. 2021 Apr 15;75(4):167–78. doi: 10.1007/s10858-021-00365-x (PMC8047523; doi:10.1007/s10858-021-00365-x)
Supplement: Supplementary file 1 — Supplementary figures (PDF 5271 kb) [file 10858_2021_365_MOESM1_ESM.pdf]

A COVID Moonshot: assessment of ligand binding to the SARS-CoV-2 main protease by saturation transfer difference NMR spectroscopy

Kantsadi A.L., Cattermole E., Matsoukas M.T., Spyroulias G.A, Vakonakis I.

Supplemental figures and captions

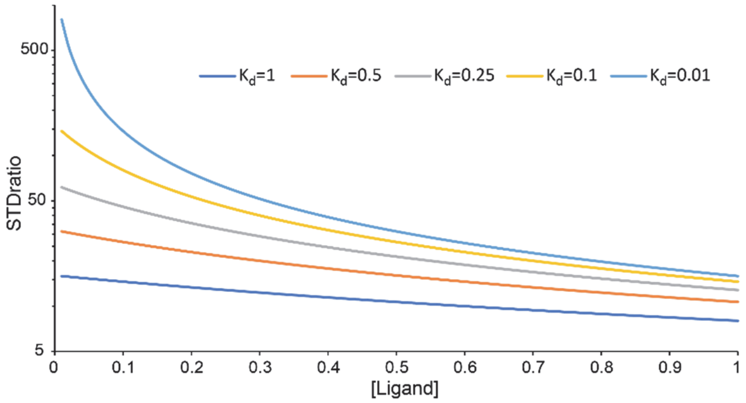

Supplemental Figure 1: STD_{ratio} values vary as function of ligand K_d and concentration. Shown here are simulations of STD_{ratio} values for arbitrary ligand concentration (0 to 1) as function of the ligand interaction strength (K_d) expressed in the same concentration scale. As seen, strongly interacting ligands (K_d of 0.1 to 0.01) produce overly large STD_{ratio} values if in low concentration, thereby skewing the correlation of STD_{ratio} values to ligand affinity.

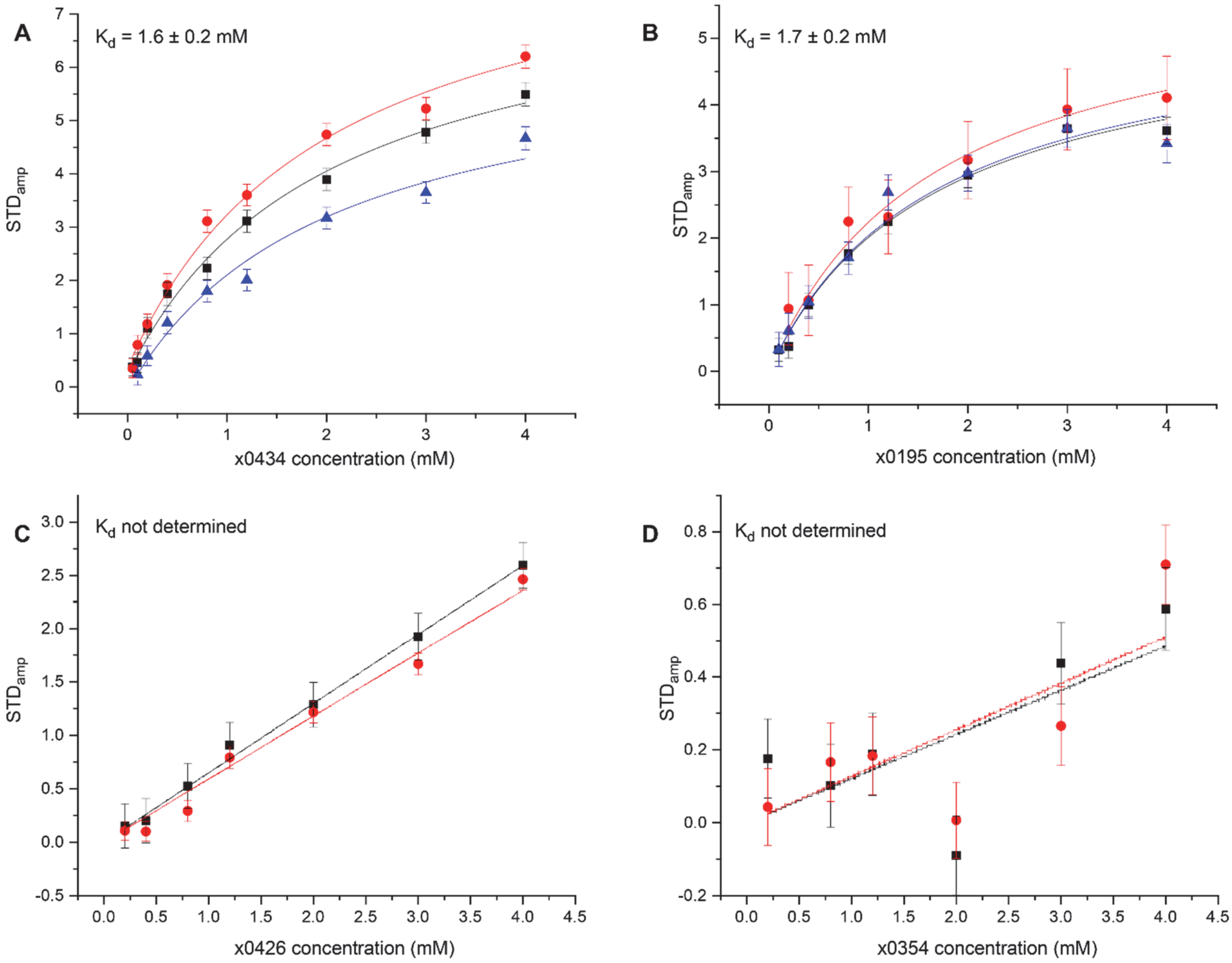

Supplemental Figure 2: Quantification of interaction affinities for chemical fragments binding to the M^{pro} active site. Shown here are plots of STD amplification factors (STD_{amp}, equal to STD_{ratio} multiplied by ligand excess) versus fragment concentration, measured in STD-NMR experiments of fragments with M^{pro}. Each data series within panels A-D corresponds to STD_{amp} values derived from separate resonance peaks of the indicated ligands. Data were fit to a single-site association model with global K_d for each ligand. As seen, whereas ligands x0434 and x0195 produced plots that could be fit to extract K_d values, the affinities of ligands x0426 and x0354 could not be determined.

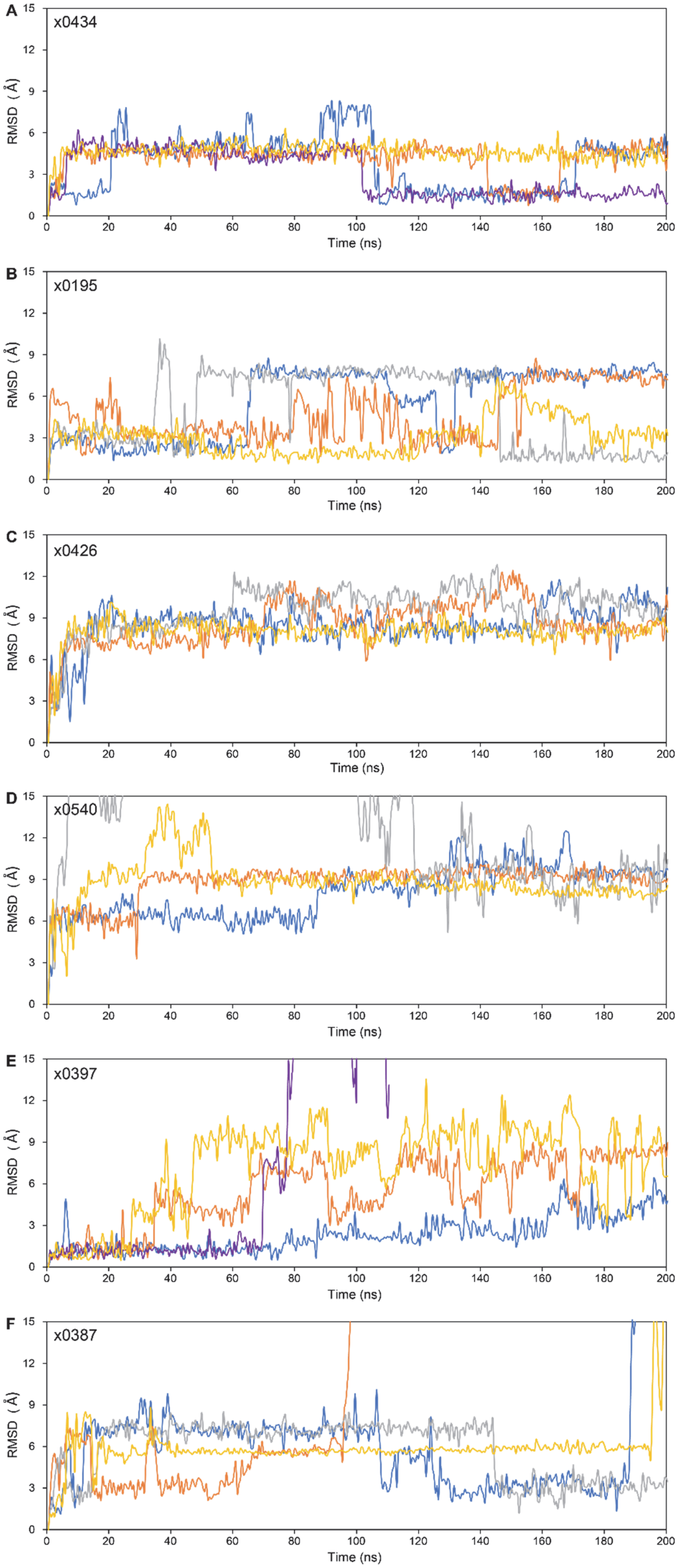
A
x0434
B
x0195
C
x0426
D
x0540
E
x0397
F
x0387
RMSD (Å)
Time (ns)

Supplemental Figure 3: MD simulations of chemical fragments binding to the M^{pro} active site. Shown here are plots of ligand RMSD from the simulation starting point as function of simulation time. Each simulation was performed four times as indicated by the different colour traces.

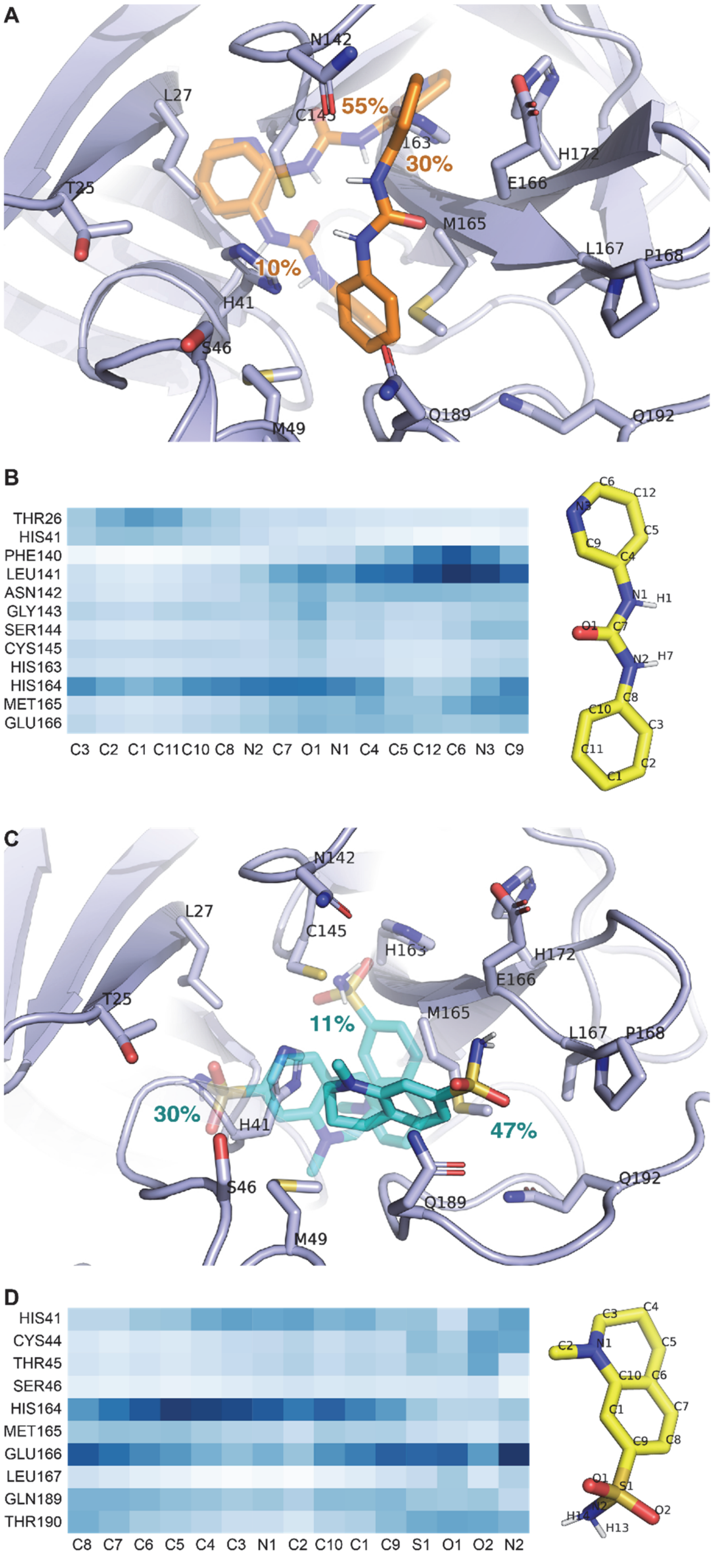

Supplemental Figure 4: Binding poses of compounds x0434 and x0195 in the M^{pro} *active site.* A) The M^{pro} active site with ligand x0434 (orange sticks) shown in the three most prevalent binding poses adopted during the quadruplicate MD simulations. The relative populations of each pose are indicated. B) Contact probability map of M^{pro} amino acids and x0434 atoms in the MD simulations. The x0434 structure and atom assignments

are shown on the right. Contact probabilities have been normalized to the most prevalent pair (darker blue shades indicating higher probability). C,D) Similar representations of the M^{pro} active site in simulations of ligand x0195 (C) and contact probability map of this ligand (D).

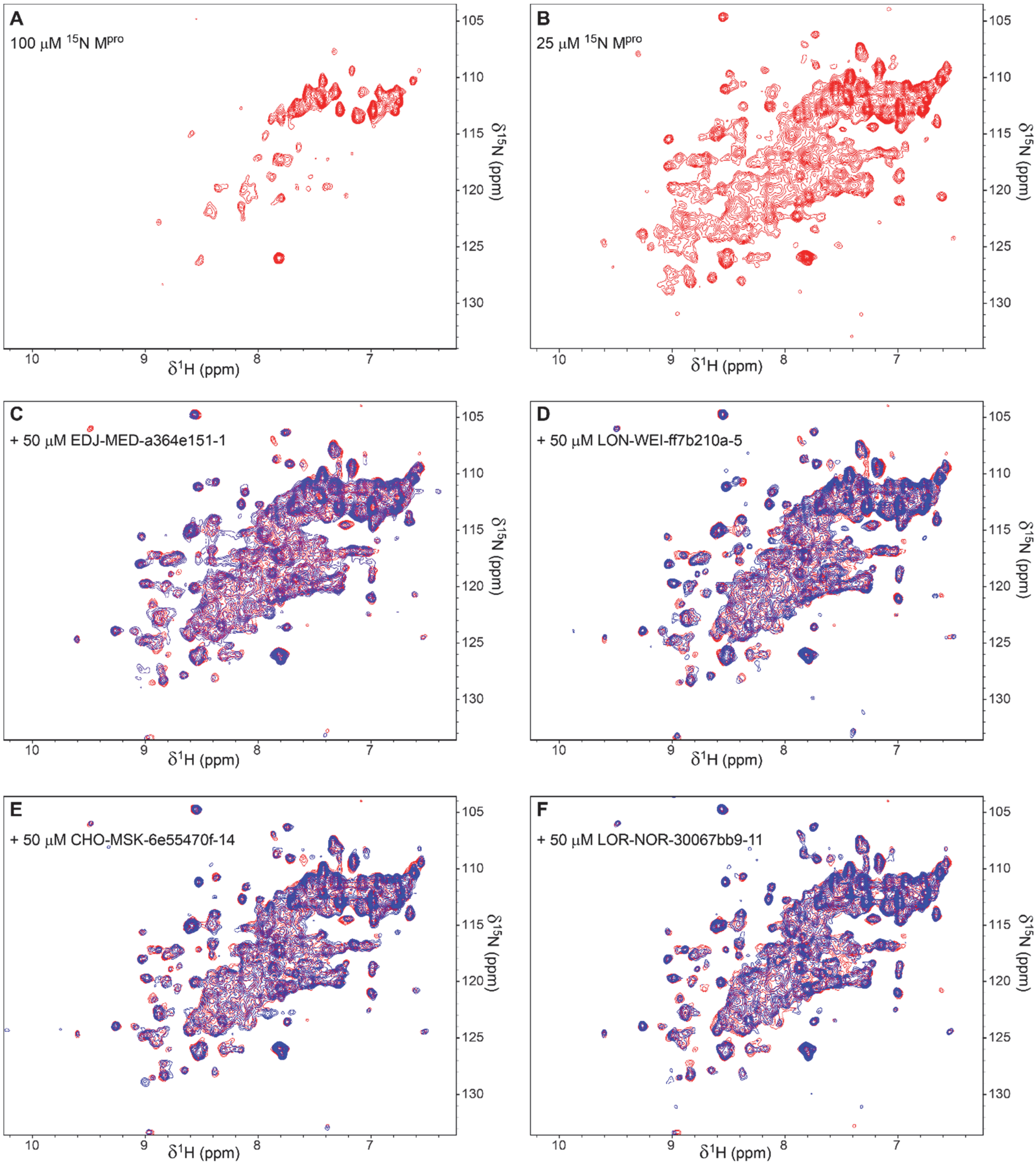

Supplemental Figure 5: 2D ^{1}H-^{15}N spectra of M^{pr}. Shown here are SOFAST-HMQC spectra of ^{15}N-enriched M^{pro} at (A) 100 μM or (B) 25 μM protein concentrations. Spectra are plotted at the same interval above their respective noise level. Spectrum (A) was recorded in approximately 2 hours (200 transients per increment of indirect acquisition) while spectrum (B) was obtained in 7.4 hours (750 transients per increment). Considering the differences in protein concentration and length of experiments, our expectation was that spectrum (A) at higher concentration would yield twice the signal-to-noise of spectrum (B); the failure to do so signifies M^{pro} aggregation at the 100 μM protein concentration level. (C-F) SOFAST-HMQC spectra of M^{pro} alone at 25 μM concentration (red) overlaid with similar M^{pro} spectra in the presence of 50 μM ligand (blue) as indicated.

Supplemental Movies S1-S6. MD simulations of chemical fragments binding to the M^{pro} active site. Movie S1 is of fragment x0434; movie S2 of fragment x0195; movie S3 of fragment x0426; movie S4 of fragment x0540; movie S5 of fragment x0397; movie S6 of fragment x0387). M^{pro} is shown in schematic representation and ligands as sticks. Hydrogen bonds are indicated by dashed yellow lines. Each pane corresponds to an independent simulation.
