# Supplementary material for: A COVID moonshot: assessment of ligand binding to the SARS-CoV-2 main protease by saturation transfer difference NMR spectroscopy
Source: J Biomol NMR. 2021 Apr 15;75(4):167–78. doi: 10.1007/s10858-021-00365-x (PMC8047523; doi:10.1007/s10858-021-00365-x)
Supplement: Supplementary file 8 — Supplementary material 7 (DOCX 134 KB) [file 10858_2021_365_MOESM8_ESM.docx]

*Supplemental Table 1: M^pro^-binding of chemical fragments assessed by STD-NMR*

| **Ligand** | **Binding site on M^pro^** | **SMILES and structure** | **STD_ratio_ (x1000)** | **Relative ligand concentration** |
| --- | --- | --- | --- | --- |
| x0072 | Active | \| CS(=O)(=O)NCCC=1C=CC=CC1 \| \| --- \| | 6.2±0.4 | 0.68 |
| x0107 | Active | \| CC(=O)NC=1C=NC=CC1C \| \| --- \| | 5.6±0.7 | 0.71 |
| x0161 | Active | \| COC(=O)C=1C=CC(=CC1)S(=O)(=O)N \| \| --- \| | 5.6±0.4 | 0.66 |
| X0165 | Surface | \| CN1CCN(CC1)C(=O)NC=2C=CC=CC2 \| \| --- \| | 0.2±0.8 | 0.29 |
| X0177 | Surface | \| NC(=O)C1CCN(CC1)C(=O)NC=2C=CC=CC2 \| \| --- \| | 4.4±0.9 | 0.27 |
| x0194 | Surface | \| CC(=O)NC=1C=CC(OC=2N=CC=CN2)=CC1 \| \| --- \| | 33±4 | 0.06 |
| x0195 | Active | \| CN1CCCC=2C=CC(=CC12)S(=O)(=O)N \| \| --- \| | 45.9±0.8 | 0.20 |
| x0305 | Active | \| CCNC=1C=CC(C#N)=CN1 \| \| --- \| | 8.7±0.8 | 0.61 |
| X0336 | Surface | \| CC(=O)NCC=1C=CC(=CC1)S(=O)(=O)N \| \| --- \| | 0.6±0.6 | 0.44 |
| X0350 | Surface | \| FC=1C=CC=C(CNCC2=CC=CO2)C1 \| \| --- \| | 12±2.5 | 0.20 |
| x0354 | Active | \| CN1CCN(CC1)C(=O)COC=2C=CC(C)=CC2 \| \| --- \| | 3.1±0.4 | 0.66 |
| X0376 | Surface | \| O=C(N1CCCCCC1)C=2C=CC=3OCOC3C2 \| \| --- \| | 3.4±0.5 | 1.00 |
| x0387 | Active | \| OC1CCN(CC=2C=CSC2)CC1 \| \| --- \| | 2.8±0.7 | 0.76 |
| x0390 | Surface | \| OC=1C=CC=CC1CNC2=NC=3C=CC=CC3N2 \| \| --- \| | 46±2 | 0.28 |
| x0395 | Active | \| CC1=NN=C(CN2CCC=C(F)C2)S1 \| \| --- \| | 3.1±0.5 | 0.99 |
| x0397 | Active | \| CN(CC=1C=C(C)ON1)C(=O)NC2CC2 \| \| --- \| | 1.8±0.7 | 0.67 |
| x0398 | Surface | \| C(CN1CCOCC1)SC=2C=CC=CC2 \| \| --- \| | 8±1.5 | 0.16 |
| x0426 | Active | \| FC=1C=CC=CC1C(=O)NCCC=2C=CN=CC2 \| \| --- \| | 17±1 | 0.21 |
| x0434 | Active | \| O=C(NC=1C=CC=CC1)NC=2C=CC=NC2 \| \| --- \| | 51±2 | 0.29 |
| x0464 | Surface | \| CC1C(O)CCCN1CC=2C=CC=CC2 \| \| --- \| | 1.0±0.2 | 0.46 |
| x0478 | Surface | \| COC(=O)C=1C=CC=C(NS(=O)(=O)C)C1 \| \| --- \| | 6.6±0.6 | 0.39 |
| X0499 | Surface | \| NC(=O)C=1C=CC(NC(=O)[C@@H]2CCCO2)=CC1 \| \| --- \| | 3±1 | 0.20 |
| x0540 | Active | \| O=C(NCCC=1C=CN=CC1)NC2CCCCC2 \| \| --- \| | 17±1 | 0.21 |
| X0669 | Surface | \| ClC=1C=CC=C(CN2CCOCC2)C1 \| \| --- \| | 8±3 | 0.16 |
| X1086 | Surface | \| CC=1C=C(F)C=CC1CS(=O)(=O)N \| \| --- \| | 27±1 | 0.45 |
| X1101 | Surface | \| CC(C)N(C)C=1N=CN=C2N(C)N=CC12 \| \| --- \| | 3±1 | 0.22 |
| X1119 | Surface | \| NC=1C=CC(=CC1)S(=O)(=O)NC=2C=CC=CN2 \| \| --- \| | 6.1±0.6 | 0.43 |
| X1132 | Surface | \| CC1CN(CCO1)C(=O)CN2C=CC=N2 \| \| --- \| | 3±1 | 0.39 |
| X1163 | Surface | \| OCC1CN(CC=2C=CC=CC2)CCO1 \| \| --- \| | 2.2±0.2 | 0.42 |
| x1187 | Dimer | \| CN1C=CC(=N1)C(=O)NC[C@@H]2CCCO2 \| \| --- \| | 7±2 | 0.30 |
| x1226 | Surface | \| CCNCC1=CN(C)N=N1 \| \| --- \| | 10±1 | 0.19 |
| X1235 | Surface | \| COC(=O)NC=1SC(C)=NC1C=2C=CC=CC2 \| \| --- \| | 10.1±0.7 | 0.38 |
| X1237 | Surface | \| CS(=O)(=O)CC1=NC=2C=CC=CC2N1 \| \| --- \| | 15.3±0.6 | 0.42 |
| x1249 | Active | \| O=C(NCC=1C=CC(C#N)=CC1)N2CCOCC2 \| \| --- \| | 4±1 | 0.25 |
